# Diverse climbing strategies in aroid vines: functional adaptations and environmental drivers

**DOI:** 10.3389/fpls.2025.1692444

**Published:** 2025-11-19

**Authors:** Pinger Yu, Shufei Weng, Bo Zhang, Yichun Huang, Feican Xu

**Affiliations:** College of Forestry and Landscape Architecture, South China Agricultural University, Guangzhou, China

**Keywords:** vines, Araceae, climbing growth, morphological plasticity, attachment strategies, environmental adaptation

## Abstract

Aroid vines are an essential group for vertical space resource utilization in tropical rainforests and serve as key materials for vertical greening in urban landscapes and home gardening. Understanding their climbing strategies is crucial for rainforest ecological conservation and alleviating urban green space shortages. However, current research on the climbing growth of aroid vines remains fragmented. This article systematically reviews the existing literature, synthesizing advances in three key research domains: functional climbing-type classification and global distribution of climbing aroids, climbing strategies, and influencing factors. The review explores the climbing strategies and morphological plasticity from the perspectives of roots, stems, and leaves. Additionally, factors such as light conditions, host surfaces characteristics, mechanical contact, and growth direction are analyzed to understand the growth traits and environmental adaptability of aroid vines. The review highlights that: (1) Aroid vines can be classified by life form into terrestrial vines, epiphytic vines, and semi-epiphytic vines, and by the degree of leaf differentiation into isomorphic, allomorphic, and heteromorphic vines. However, their current life-form classification systems remain to be refined; (2) Root adhesion serves as the core climbing strategy, characterized by the dimorphism of aerial roots, which facilitate attachment through clasping roots; (3) Aroid vines exhibit pronounced morphological plasticity to cope with environmental transitions from the terrestrial to the canopy, reallocating resources toward aboveground organs—enhancing water and nutrient transport through the proliferation of feeder roots, stem thickening, and hydraulic optimization, while promoting photosynthetic efficiency and drought tolerance via heterophyllous leaf development; (4) Negative phototropism drives the search for supports, light intensity regulates leaf morphological development, and host surface roughness and material significantly affect attachment efficiency and biomass allocation. Mechanical contact and gravity direction influence growth through hormone homeostasis, coordinating resource distribution. Based on current findings, future research directions and actionable recommendations are proposed to provide a theoretical foundation for the further exploitation and utilization of aroid vines.

## 1 Introduction

The Araceae family is one of the most morphologically and ecologically diverse groups among monocotyledonous plants, comprising 9 subfamilies, and 114–144 genera, with approximately 3600–4500 species worldwide (Wu, 2010; Boyce and Croat, 2020; Croat, 1979; International Aroid Society, 2025). Recent years have also seen the description of new species (Valadares et al., 2024; Collantes et al., 2024). The Araceae family exhibits a cosmopolitan distribution, with its peak diversity concentrated in humid tropical regions. Araceae plants exhibit various life forms, including terrestrial, epiphytic, semi-epiphytic, and aquatic types (Croat and Ortiz, 2020), with many being vines (Zuluaga et al., 2019). Aroid vines are a key group for utilizing vertical space resources in tropical rainforests, making significant contributions to forest community structure and essential ecological processes. They play important roles in facilitating secondary succession and regulating carbon, water, and nutrient cycles (Ward et al., 2020), while accelerating the formation of vertical forest structure, altering competitive dynamics within stands, and influencing host tree performance (Verbeeck et al., 2024). Moreover, they provide food resources and habitats for animals, thereby contributing to the maintenance of ecological diversity (Schnitzer et al., 2020). In addition, due to their unique and diverse leaf shapes, colors, and excellent shade-tolerant growth characteristics, aroid vines have become an important choice for modern urban vertical greening projects and indoor plant landscaping (Henny et al., 2004). They offer various ecological and socio-economic benefits, including increasing urban vertical greening coverage, improving thermal environments, and alleviating anxiety among city dwellers (Meng et al., 2022; Chatakul and Janpathompong, 2022).

In recent years, research on aroid vines has focused on climbing strategies, genomic evolution (Jia et al., 2025; Xu et al., 2024), vertical greening, and ecological applications (Wang et al., 2018). Some research also focused on resource survey (Zhang et al., 2017; Othman et al., 2010), and tissue culture propagation (Yang et al., 2018). However, there is still a lack of systematic reviews and synthesis of their climbing strategies and environmental response strategies. This article provides a summary of the global distribution and classification, climbing strategies, and influencing factors of aroid vines. It aims to address the following questions: (a) Which types can aroid vines be classified based on differences in climbing traits? (b) What strategies do aroid vines employ to achieve attachment? (c) How do these plants adapt to the environmental changes associated with climbing? and (d) Which environmental factors influence their climbing growth, and through what strategies do they exert their effects? Furthermore, the review proposes future research directions to provide a theoretical basis for the further development and utilization of aroid vines.

## 2 Materials and methods

Relevant literature was retrieved from multiple academic databases, including Scopus, PubMed, Web of Science, EBSCO Green FILE, Google Scholar, and CNKI, using the keywords “Aroid”, “Climbing Mechanisms”, “Araceae”, “Tropical Forest”, “Attachment Mechanism”, and “Clinging Climber.” The collected publications were carefully examined within the context of climbing strategies in Araceae Vines, and a subset of representative studies was further selected for in-depth analysis and synthesis.

## 3 Diversity and distribution of aroid vines

### 3.1 Geographic ranges of aroid vines

Araceae plants are highly concentrated in tropical regions. In the Neotropics, approximately 8 genera and 425 species of Araceae have been reported as vines (Smithsonian National Museum of Natural History, 2025). The Pacific coastal area of the Northern Andes is a hotspot for species diversity (Herrera et al., 2008). Approximately two-thirds of the species are distributed across the tropical regions of South America (Croat, 1979), such as in the Amazon basin, which is home to moisture-tolerant species like *Monstera adansoni*i (Schnitzer and Bongers, 2002). In Central America, the distribution of aroid vines is closely related to temperature and humidity, with species diversity peaking in humid low- to mid-elevation areas in Ecuador (Croat, 1992, 1995). In the Old World tropics, Southeast Asia, particularly the Malay Archipelago, is another diversity center, with Borneo recording 12 genera and 80 species (Putz, 1984). In China, there are 10 endemic genera and 71 species, distributed across 26 provinces, with the highest diversity found in Yunnan Province (Liang et al., 2019).Additionally,Substantial distributions are also observed in tropical African regions such as Madagascar (Henriquez et al., 2014).

Araceae species are widely distributed across diverse habitats, with warm and humid tropical forests representing their primary habitat. This includes numerous species of *Anthurium*, *Aglaonema*, *Homalomena*, *Monstera*, *Philodendron* and *Rhaphidophora* (Boyce and Croat, 2020). In aquatic habitats, the family is represented by the subfamily Lemnoideae, which float on the water surface, or by genera such as *Cryptocoryne*, which are rooted and grow submerged. In swampy habitats, genera such as *Lysichiton* and *Zantedeschia* occur in shallow waterlogged areas (Croat, 1988). In seasonally dry forests, genera including *Amorphophallus*, *Dracontium*, and *Zamioculcas* adapt to drought through enlarged rhizomes (Holtum et al., 2007). Limestone habitats are typified by genera such as *Homalomena* and *Schismatoglottis* (Boyce and Wong, 2009, 2019), whereas secondary open habitats are occupied by genera including *Xanthosoma* and *Epipremnum* (Croat and Ortiz, 2020).

### 3.2 Functional climbing-type classification

Aroid vines are primarily distributed in the subfamilies Monsteroideae and Pothoideae (Zhao et al., 2023). The core genera in Monsteroideae include *Epipremnum*, such as *Epipremnum pinnatum*; *Monstera*, such as *Monstera deliciosa*; *Philodendron*, (Loss-Oliveira et al., 2016) such as *Philodendron hederaceum*; *Scindapsus*, such as *Scindapsus maclurei*; and *Rhaphidophora*, such as *Rhaphidophora decursiva*. The core genera in Pothoideae include *Pothos*, such as *Pothos chinensis*; *Pothoidium*, such as *Pothoidium lobbianum*; and *Pedicellarum*, such as *Pedicellarum paiei* (Cusimano et al., 2011).

Aroid vines can be categorized based on their life form (**Table 1**), including terrestrial, epiphytic, and semi-epiphytic types. Terrestrial vine can be divided into terrestrial climbing and terrestrial creeping types. Most aroid vines exhibit a terrestrial climbing habit, although some species possess weak climbing abilities and primarily grow prostrate, representing the terrestrial creeping type. Examples include certain species of *Scindapsus* and *Philodendron* (Saibeh, 2010; Vasconcelos et al., 2018). Kress and Putz categorized semi-epiphytic plants into “primary semi-epiphytic” and “secondary semi-epiphytic” plants (Kress, 1986; Putz and Holbrook, 1986), with the latter still lacking empirical support. Moffett and Zotz proposed the term “nomadic vines” to replace “secondary semi-epiphytic” plants (Moffett, 2000; Zotz, 2013). In field studies, few species clearly fit the semi-epiphytic classification, particularly those classified as nomadic vines, as such categories often do not align with their *in situ* life histories, suggesting the need for further data on the classification of aroid vines’ life forms (Einzmann et al., 2024).

**Table 1:** Life form classification of aroid vines.

Category		Germination site	Method of plant-soil connection	Representative species	References
Terrestrial vine	Terrestrial climbing vine	Ground	Permanent stem-soil connection	*Epipremnum aureum*	(Croat and Ortiz, 2020)
	Terrestrial creeping vine	Ground	Permanent stem-soil connection	*Scindapsus pictus*	(Saibeh, 2010)
Epiphytic vine		Host surface	Permanently epiphytic, no soil contact	*Anthurium scandens*	(Croat and Ortiz, 2020; Kress, 1986; Putz and Holbrook, 1986)
Semi -epiphytic vine	Primary Semi -epiphytic vine	Host surface	Initial epiphytic growth. soil connection established later via adventitious roots	*Anthurium clavigerum*	(Kress, 1986; Putz and Holbrook, 1986)
	Nomadic vines	Ground	Initial stem-soil connection, but as the plant matures, the connection to the soil decreases. In the later stages, soil contact maintained via adventitious roots	*Monstera dubia*	(Moffett, 2000; Zotz, 2013; Einzmann et al., 2024)

**Figure 1:**
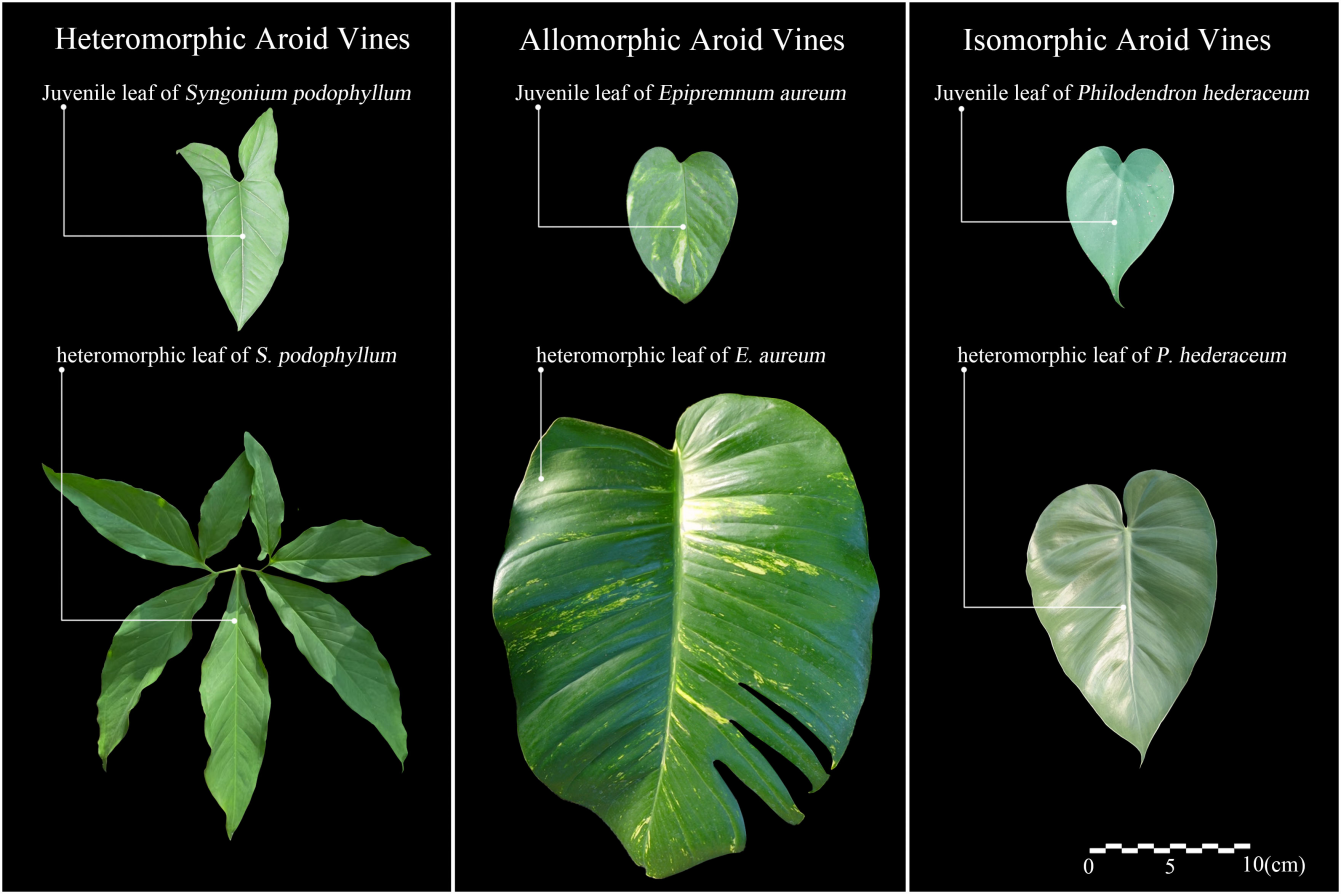
Classification of leaf morphological variation in aroid vines.

Based on the degree of woodiness at the base, climbing plants can be classified into two types: herbaceous vines and woody vines. According to their climbing modes and climbing organs, Darwin divided climbing plants into four categories: stem twining, tendril twining, hook or spine attachment, and root climbing (Darwin, 1865). On this basis, Putz further classified them into tendril adhesion and root climbing (Putz, 1984). Isnard and Silk grouped root adhesion and suction disk adhesion under the general term of root-attaching vines (Isnard and Silk, 2009). Sperotto and others further consolidated these categories into two major types: active and passive climbers. Active climbers include stem twining, tendrils, climbing branches, coiling petioles, and flower clusters, while passive climbers include simple attachment, hooks or anchor structures, and adhesive roots (Sperotto et al., 2020). Most aroid vines are herbaceous vines, belonging to the root-attaching vines, and rely on adventitious roots for climbing, classifying them as passive climbers. More specifically, based on leaf morphological variation during their climbing growth, Ray categorized aroid vines into three types (**Figure 1**), including heteromorphic (significant changes in both leaf size and shape), allomorphic (significant changes in leaf size but minor changes in shape), and isomorphic (minor changes in both size and shape) (Ray, 1990).

## 4 Adaptations and functional traits of aroid vines for climbing

**Figure 2:**
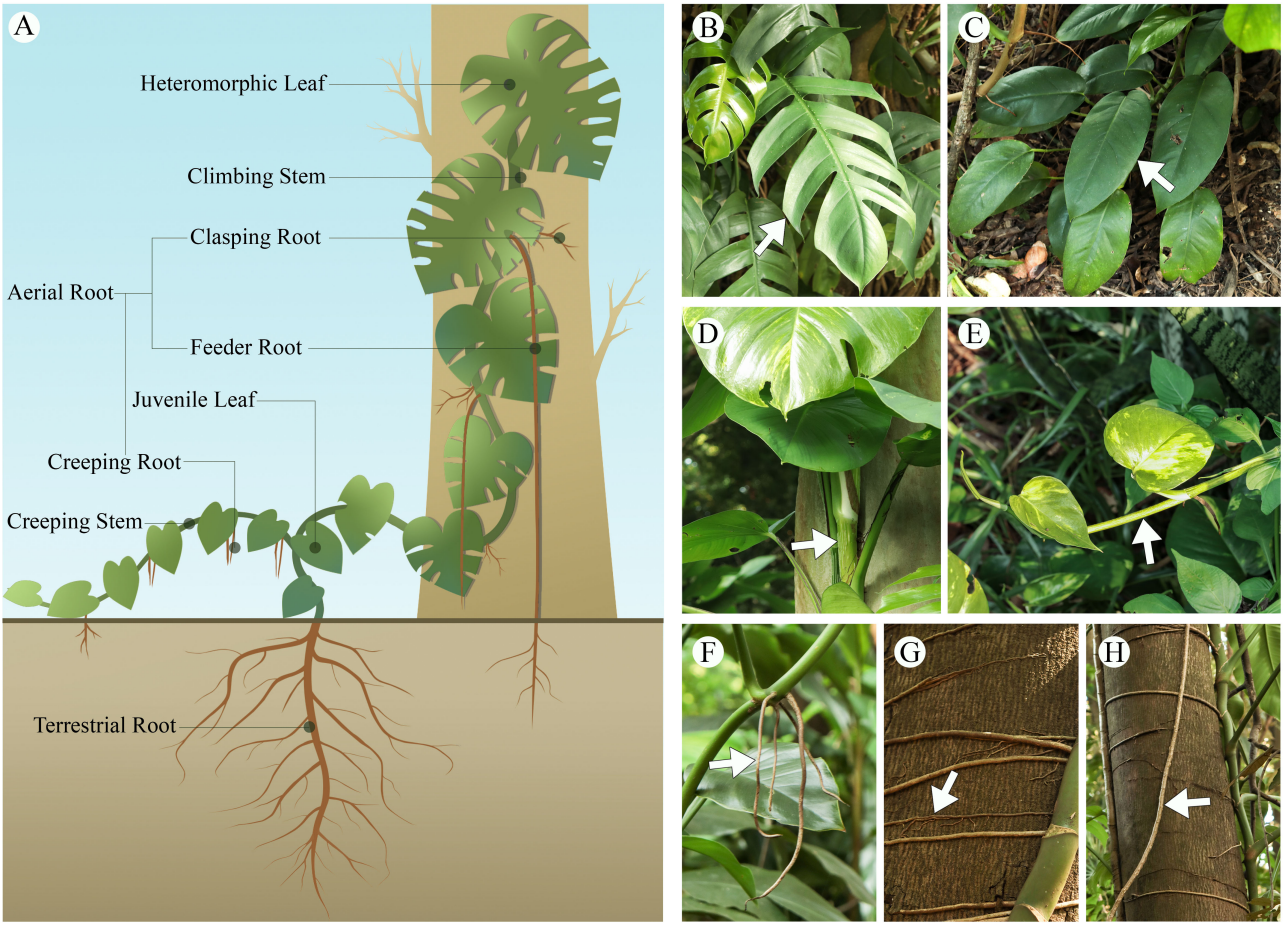
Morphological adaptations of aroid vines in growth. **(A)** illustrative diagram of aroid vines growth. **(B)** heteromorphic leaf of *Epipremnum pinnatum*, **(C)** Juvenile leaf of *E. pinnatum*, **(D)** climbing stem of *Epipremnum aureum*, **(E)** creeping stem of *E. aureum*, **(F)** creeping root of *Rhaphidophora megaphylla*, **(G)** clasping root of *R. megaphylla*, **(H)** feeder root of *R. megaphylla*.

Aroid climbing vines undergo both creeping and climbing stages during their growth; in response to environmental changes, their roots, stems, and leaves exhibit distinct morphological and structural characteristics at each stage (**Figure 2**). These adaptive growth strategies are crucial for maintaining physiological function and ensuring population persistence in the dynamic conditions of tropical rainforest environments.

### 4.1 Root adaptations

Aroid vines are classified as root-attaching vines, possessing both underground and aerial roots. The aerial roots often undergo color changes as they mature; for instance, in *Rhodospatha oblongata*, roots transition from reddish-brown to streaked and eventually to green, containing chloroplasts that enable additional light capture to support growth (Filartiga et al., 2021). Specifically, When aerial roots come into contact with the host surface, they are densely covered with root hairs, whereas those exposed to the air are hairless (Filartiga et al., 2014). Some of these aerial roots undergo morphological changes when transitioning to different habitats (French, 1987)(**Table 2**; **Figures 2E–G**). In creeping growth, the aerial roots are typically short and fine, but once the plant attaches to a support, these roots differentiate into two types: feeder roots that absorb water and nutrients from the host surface, and clasping roots that anchor the plant to the host (Putz and Mooney, 2011). These two types of roots exhibit functional and morphological dimorphism.

**Table 2:** Strategies of root growth in aroid vines.

Growth mode	Plant organ	Morphological characteristics	Structural characteristics	Ecological function	References
Creeping growth	Creeping root	The roots are short and thin, unbranched, growing vertically downward, white in color, turning brown with growth.	Narrow stele, fewer and smaller vessels, sparse root hairs.	Potential to transform into climbing or feeder roots; elongates downward to support vegetative propagation and nutrient absorption.	(Putz and Mooney, 2011; Filartiga et al., 2014; Yang and Deng, 2017)
Climbing growth	Clasping roots	the roots are longer and thicker than creeping roots, but shorter and thinner than nutrient roots, which grow laterally and branch easily, with a smaller diameter.	Thicker stele, larger and more numerous vessels, contains chloroplasts, thinner secondary DTB, lower lignification, presence of specialized root hairs, secretes mucilage.	Weak photosynthetic capacity; secretes polysaccharide-protein mucilage for adhesion; does not contact soil, stabilizes attachment to host.	
	Feeder roots	The roots are the longest among all root types, unbranched, with the largest diameter, growing vertically downward until they contact the soil and transform into terrestrial roots.	Thickened stele, increased vessel size and number, contains chloroplasts, thick secondary DTB, higher lignification; retains trichomes and cortex on host-contact side, develops cork layer on exposed side.	Relatively high photosynthetic capacity; connects canopy to soil for water and nutrient transport; forms an efficient water transport system.	

Studies have shown that feeder roots grow downward to reconnect with the soil, possessing larger vessel diameters and a thickened suberized double thickened band layer, which confer greater hydraulic efficiency and water retention capacity compared with other root types (Filartiga et al., 2014). In contrast, clasping roots grow laterally, and their development typically proceeds through three distinct stages: first, aerial roots emerge at the node; then, the root cap secretes mucilage to assist initial adhesion upon contact with the support; and finally, the roots elongate and develop root hairs, adjusting their morphology according to the host surface texture, secreting a polysaccharide-protein composite gel to enhance adhesion. For instance, *Syngonium podophyllum* produces roots in a specific pattern, with rapid root growth in the early stage, followed by slower growth after root formation. The strength of adhesion increases over time. In addition, *Syngonium possesses* unique spirally-splitting root hairs that enhance adhesion, and their formation is influenced by external mechanical forces (Yang and Deng, 2017).

The differentiation of root systems in aroid vines represents an adaptive response to the increasing demands for mechanical support and water–nutrient supply during climbing growth. As the plant ascends, both the number and size of clasping and feeder roots increase proportionally with height (Filartiga et al., 2014). The proliferation of clasping roots enhances the plant’s overall adhesion strength, ensuring stable attachment as biomass accumulates. In contrast, feeder roots are typically longer and thicker than clasping roots and maintain direct connection with the soil, enabling efficient compensation for the elevated hydraulic resistance associated with longer stems and providing additional water and nutrient support for continued vertical growth.

### 4.2 Stem adaptations

Aroid vines typically have succulent stems, with some species having few branches. Vines often exhibit polymorphic stems, which include both creeping and climbing types (Metcalfe, 2005). During both creeping and climbing growth, the stems of aroid vines show characteristics of both types (**Table 3**; **Figures 2C, D**). Creeping stems of aroid vines are typically characterized by shorter internodes and smaller diameters, allocating a greater proportion of resources to leaf development and exhibiting heightened light sensitivity. In contrast, climbing stems generally possess longer and thicker internodes and develop specialized structures such as aerial roots to aid in attachment to supporting substrates. During the initial climbing phase, resources are primarily allocated to stem elongation, often accompanied by delayed leaf expansion (Cai and Song, 2001; Mantovani et al., 2018; Wyka, 2023). Unlike many other vines, however, creeping and climbing stems in aroid vines typically occur sequentially along the same axis, reflecting a gradual developmental transition rather than distinct structural forms. Furthermore, as the attachment height increases, the stele diameter of climbing stems enlarges, accompanied by an increase in both the number and size of xylem vessels (Filartiga et al., 2014). In contrast to the tapering xylem model observed in trees (West et al., 1999), this pattern forms an “inverted cone” structure, resembling that of *Monstera acuminata* (López−Portillo et al., 2000).

**Table 3:** Strategies of stem growth in aroid vines.

Growth mode	Plant organ	Morphological characteristics	Structural characteristics	Ecological function	References
Creeping growth	Creeping stem	Smaller diameter, short internodes with relatively uniform length.	Larger stele diameter, fewer and smaller xylem vessels; active parenchyma at nodes with well-developed meristematic tissue.	Ensures water and nutrient transport, facilitates ground-level expansion and vegetative propagation; explores for support in shaded environments.	(Metcalfe, 2005; Kato et al., 2012b; Wyka, 2023)
Climbing growth	Climbing stem	Thicker stem, longer internodes; exhibits allometric growth; internodes shorten after reaching canopy.	Larger stele diameter, more and larger xylem vessels.	Facilitates water and nutrient transport, anchors to support structures, maintains upright growth, expands vertical spatial range.	(Filartiga et al., 2014; Cai and Song, 2001; Kato et al., 2012b; Wyka, 2023; Mantovani et al., 2018; Ray, 1986)

Aroid vines also exhibit allometric growth in the stems during climbing. Early in the climbing process, internode elongation facilitates rapid extension to capture more light. Once an optimal height is reached, internodes shorten and thicken to slow vertical growth (Ray, 1986), thereby reallocating resources toward the production of larger and more numerous leaves. Some aroid vines also develop searching shoots with long internodes and rapid growth. These searching shoots possess movement ability, spiraling around the support by changing the shape and volume of epidermal cells and actively finding a suitable support to climb (Putz and Mooney, 2011). Once the plant has reached the optimal height, the searching shoots help the plant spread to other parts of the same tree or even different trees to exploit new resources. For example, *Philodendron linnaei* is highly adept at repositioning itself in response to environmental conditions. It can repeatedly return to the ground, seek out new supports to climb, and simultaneously adjust its stem, petiole, and leaf morphology to meet the demands of a continuously changing environment (Ray, 1981; 1987; 1989).

The heteromorphic stems of aroid vines reflect distinct growth strategies at different developmental stages. Creeping stems primarily function in vegetative growth and lateral expansion within the forest understory to locate support structures, whereas climbing stems focus on attaching to supports and developing vertical structures to ensure hydraulic function (Kato et al., 2012b). Stem hydraulic optimization is achieved by increasing the number and grouping of vessels to compensate for the hydraulic resistance incurred by increased stem length. This strategy not only enhances water transport efficiency but also reduces the risk of embolism by introducing more and larger vessels (Zimmermann, 1983), thereby supporting the maintenance of large plant stature. Notably, during the climbing process, the rate of increase in the cross-sectional area of the stele exceeds that of vessel number (Filartiga et al., 2014), potentially reducing hydraulic efficiency per unit stele area. This pattern may reflect a trade-off between mechanical support and hydraulic transport.

### 4.3 Leaf adaptations

During the transition from creeping to climbing growth, aroid vines undergo a series of leaf morphological changes (**Table 4**; **Figures 2A, B**). Many aroid vines develop heterophylly, developing juvenile leaves during early growth stages and mature leaves during the adult phase (Nakayama, 2024; Mou et al., 2019). Heterophyllous leaf development is typically a gradual process that occurs progressively during the ascent from the forest floor to the upper canopy. Anatomically, leaf traits such as leaf area, leaf thickness, adaxial cuticle thickness, mesophyll thickness, palisade parenchyma thickness, spongy parenchyma thickness, abaxial cuticle thickness, stomatal density, stomatal pore height, and guard cell size generally increase with climbing height (**Table 5**). The petiole also becomes longer and thicker, with an increased vessel diameter in the xylem (Filartiga et al., 2018). This indicates a significant positive correlation between leaf and petiole traits in aroid vines. These plants tend to develop thicker leaves and epidermis, along with higher stomatal density, to reduce water loss and adapt to environments characterized by high light intensity and low humidity (Ardiningtyas et al., 2023). In addition, canopy leaves exhibit extended lifespans, ranging from 28 to 40 months (Shiodera et al., 2008). In terms of photosynthetic physiology, leaves in the upper canopy generally exhibit significantly higher levels of chlorophyll a, chlorophyll b, total chlorophyll, maximum electron transport rate, photosynthetically active radiation at maximum ETR, assimilation rate at light saturation, and total leaf nitrogen compared to lower canopy leaves (Mantovani et al., 2018, 2017; Mantuano et al., 2021; Lorenzo et al., 2009). However, in the absence of a supporting structure for climbing, plants may remain in a juvenile state indefinitely (Filartiga et al., 2021).

**Table 4:** Strategies of leaf growth in aroid vines.

Growth mode	Plant organ	Morphological characteristics	Structural characteristics	Ecological function	References
Creeping growth	Juvenile leaf	Smaller leaf area, less lobing, thinner blade, shorter and thinner petiole.	Lower stomatal density, mesophyll thickness, vein area, and chlorophyll content; denser spongy mesophyll with smaller intercellular spaces; thinner xylem vessels in petiole.	Reduces energy consumption during seedling stage, enhances survival rate, and aids in horizontal expansion.	(Ray, 1981; Shiodera et al., 2008; Mantovani et al., 2017; Filartiga et al., 2018; Liu et al., 2016; Moodley et al., 2017)
Climbing growth	Heteromorphic leaf	Larger leaf area, increased length-to-width ratio and thickness, leaf margins develop lobes or splits; petiole longer and thicker.	Higher stomatal density, mesophyll thickness, vein area, and chlorophyll content; more developed spongy tissue with larger intercellular spaces; optimized nitrogen allocation; thicker xylem vessels in petiole.	Maximizes photosynthetic area, enhances light capture, maintains carbon and light gain efficiency.	

**Table 5:** Anatomical trends of aroid vines leaves.

Leaf anatomical traits	*Philodendron hederaceum* (Mantovani et al., 2018)			Trend	*Epipremnum aureum* (Mantovani et al., 2017)			Trend	*Rhodospatha oblongata* (Mantuano et al., 2021)			Trend	*Anthurium scandens* (Lorenzo et al., 2009)			Trend
	Terrest -rial (0m)	Lower canopy (1.5m)	Higher canopy (3m)		Terrest -rial (0m)	Lower canopy (1.5m)	Higher canopy (6m)		Terrest -rial (0m)	Lower canopy (3m)	Higher canopy (6m)		Terrest -rial (1.5m)	Lower canopy (2.25m)	Higher canopy (3m)	
Leaf area (cm2)	52.48 ± 11.56	96.40 ± 26.49	176.50 ± 69.73	↑	53.18 ± 3.67	161.94 ± 22.85	1886.59 ± 345.83	↑	29.63 ± 3.42	461.19 ± 112.20	691.90 ± 46.47	↑	data not available			↑
Leaf thickness (mm)	0.35 ±0.007	0.35 ± 0.03	0.35 ± 0.03	↔	0.388 ± 0.012	0.449 ± 0.024	0.506 ± 0.012	↑	0.150 ± 0.237	0.149 ± 0.194	0.182 ± 0.365	↑	0.269 ± 0.243	0.385 ± 0.504	0.424 ± 0.349	↑
Leaf succulence (10-3g/cm2)	79.75 ± 2.04	78.98 ± 2.92	76.43 ± 3.09	↓	28.52 ± 0.85	35.06 ± 1.09	39.20 ± 0.93	↑	N.A.				N.A.			
Adaxial epidermis thickness (μm)	58.32 ± 2.88	70.48 ± 16.41	58.32 ± 5.05	↑↓	62.99 ± 3.38	75.91 ± 2.75	72.77 ± 1.63	↑	N.A.				64.7 ± 7.2	43.8 ± 7.2	40.1 ± 5.2	↓
Adaxial cuticle thickness (μm)	N.A.				6.22 ± 0.58	8.74 ± 0.30	10.36 ± 0.23	↑	N.A.				3.0 ± 0.7	5.0 ± 1.4	6.7 ± 1.3	↑
Mesophyll thickness (μm)	N.A.				269.50 ± 13.47	322.67 ± 21.99	339.90 ± 2.14	↑	N.A.				151.3 ± 27.2	300 ± 48	340.4 ± 35.8	↑
Palisade parenchyma thickness (μm)	N.A.				56.80 ± 2.60	59.57 ± 4.11	68.31 ± 0.94	↑	40.17 ± 2.30	32.77 ± 5.85	48.24 ± 0.94	↑	96.2 ± 9.5	103.6 ± 14	123.9 ± 11.2	↑
Spongy parenchyma thickness (μm)	N.A.				187.73 ± 10.12	218.17 ± 10.98	276.10 ± 4.36	↑	50.81 ± 2.98	71.67 ± 12.36	102.94 ± 6.64	↑	100.8 ± 24.3	203.7 ± 38.8	236.6 ± 19.4	↑
Abaxial epidermis thickness (μm)	51.60 ± 5.89	59.40 ± 15.22	57.84 ± 13.32	↑	58.43 ± 1.36	63.46 ± 4.71	55.91 ± 11.67	↑↓	N.A.				49.9 ± 5.9	35.2 ± 3.1	27.7 ± 6.2	↓
Abaxial cuticle thickness (μm)	N.A.				6.39 ± 0.62	6.79 ± 0.22	9.59 ± 0.12	↑	N.A.				2.7 ± 0.7	4.0 ± 1.2	6.4 ± 0.8	↑
Stomatal density (mm-2)	4.05 ± 0.91	5.05 ± 1.25	6.48 ± 0.63	↑	N.A.				42 ± 9	53 ± 10	65 ± 20	↑	25.6 ± 3.6	32.8 ± 9.1	29.5 ± 10.3	↑
Stomatal pore height (μm)	N.A.				33.54 ± 2.93	41.14 ± 0.98	44.57 ± 1.83	↑	N.A.				35.3 ± 5.2	36.4 ± 5.2	35.3 ± 8.0	↔
Guard cell width (μm)	N.A.				9.17 ± 1.00	10.31 ± 0.97	11.83 ± 1.27	↑	N.A.				17.2 ± 3.2	16.1 ± 3.2	18.2 ± 3.8	↑

Arrows indicate the direction of trait change: ↑, increase; ↓, decrease; ↔, no clear change; ↑↓, increase followed by decrease. N.A., data not available.

Leaf adaptive strategies in aroid vines vary among species. In fully epiphytic species such as *Anthurium scandens*, canopy ascent is accompanied by marked leaf area expansion, while leaves remain thin, the chlorophyll a/b ratio declines, and chlorophyll content remains high, reflecting a strategy of compensating for low light through leaf area expansion while maintaining a shade-adapted photosynthetic system. In isomorphic species such as *Philodendron cordatum* and *Rhodospatha oblongata*, leaf thickness increases significantly with height despite the relatively small increase in leaf area, alongside enhanced palisade thickness and stomatal density, increased chlorophyll a/b ratio, and higher photosynthetic rates (Shiodera et al., 2008), indicating strong photosynthetic plasticity and adaptation to both shade and sun environments. In allomorphic species such as *Epipremnum aureum*, heterophyll size can exceed juvenile leaves by up to 30-fold (Mantovani et al., 2017), with increased length-to-width ratio, development of marginal lobes or fenestrations (Ray, 1981), and progressive increases in leaf thickness, mesophyll thickness, and palisade parenchyma thickness with height. Leaf nitrogen concentration and area-based nitrogen content also increase markedly, reflecting a strategy for efficient high-light utilization, achieved via mesophyll thickening and enhanced nitrogen allocation to photosynthetic proteins. In heteromorphic species like *Syngonium podophyllum*, seedlings produce sagittate leaves that gradually develop into larger palmate leaves with up to seven or more lobes, though detailed anatomical and physiological changes during climbing remain unquantified.

Heteroblastic leaf development is common in aroid vines and represents an adaptive foraging strategy to cope with environmental changes encountered during ascent from the forest floor to the upper canopy. This process enhances light capture and supports reproductive growth (Mantuano et al., 2021). Under energy-limited, shaded understory conditions, plants produce small juvenile leaves to reduce metabolic costs and increase survival while searching for potential hosts. Juvenile leaves often exhibit high photosynthetic potential, enabling rapid acclimation to disturbed habitats such as forest edges or high-light gaps (Moodley et al., 2017). Upon successful climbing, leaf development progressively approaches a heteroblastic form, culminating in mature heterophylls in the upper canopy. These leaves increase in size and thickness, and their lifespan increases, indicating a shift in the plant’s growth strategy from a resource-acquisitive form to a resource-conservative form. Accompanying anatomical and photosynthetic changes enhance photosynthetic capacity, water retention (Lorenzo et al., 2009), and carbon acquisition efficiency, thereby optimizing the trade-off between light capture and carbon fixation and enabling mature leaves to thrive under the high-light and dry conditions of the tropical forest canopy.

## 5 Environmental drivers of climbing growth in aroid vines

### 5.1 Light availability and intensity

Light serves as both a stimulus and a directional cue for the climbing growth of aroid vines. Plants typically exhibit phototropism, where organs bend and grow toward light (Zhang et al., 2018). Light, as an external environmental cue, stimulates plants to alter their growth direction. When light is unevenly distributed, plant shoots change growth patterns to reposition themselves for optimal light intensity (Rodriguez-Quintero et al., 2022). Aroid vines also exhibit negative phototropism (Kato, 2019), where they grow away from the light source, often during the juvenile stage when the stem grows toward the darkest area of the horizontal plane. Once a support is encountered, the stem tip bends upward, and growth is directed toward the light (Kato, 2019). This negative phototropism helps the plant more efficiently locate support structures in low-light environments, such as forest floors where shadows are cast by large trees, rocks, and cliffs (Strong and Ray, 1975). Negative phototropism is enhanced in shaded environments and suppressed under high light intensity (Rodriguez-Quintero et al., 2022; Wyka, 2023). For instance, *Syngonium podophyllum* exhibit pronounced negative phototropic behavior, growing toward darker areas to adhere to supporting structures (Yang and Deng, 2017). However, phototropism and negative phototropism are not mutually exclusive strategies—they may occur in different branches of the same plant (Kato et al., 2012a), or even at different developmental stages along the same branch (Kato, 2019).

Light is a critical factor influencing the phenotype of aroid vines (Wang P. et al., 2022), promoting plant development and leaf enlargement (Fritz et al., 2018). Once climbing is achieved, plants gain access to increased light availability, necessitating physiological adjustments to cope with light stress and optimize light utilization. Under high-light conditions, aroid leaves generally become larger and thicker, specific leaf area decreases, and spongy mesophyll develops more extensively. This structure, characterized by larger intercellular air spaces, enhances the capacity for carbon assimilation and light capture (Mantuano et al., 2021). In heteroblastic species, sun-exposed leaves additionally develop larger perforations and higher stomatal density, facilitating heat dissipation and improving thermoregulation under high irradiance (Díaz-Valverde et al., 2025). For example, in *Epipremnum aureum*, leaves exposed to high light in climbing conditions are four times larger compared to those in low light, and this growth is accompanied by thicker petioles and stems. In contrast, under low light, *E. aureum* adopts a “shade-avoidance” strategy (Poorter and Rozendaal, 2008), characterized by slender petioles and short internodes, with no significant differences in leaf area among different growth orientations. This reflects its adaptation to low-light environments and suggests that sufficient light availability is a necessary condition for leaf expansion in aroid vines (Brito et al., 2022).

### 5.2 Characteristics of host surface

The characteristics of the host surface play an important role in the climbing growth and morphological plasticity of aroid vines. As root-attaching vines, they are not limited by the size of the support and can attach to supports of various diameters and materials (Paul and Yavitt, 2011; Durigon et al., 2013). Different species generally exhibit distinct host preferences (Callaway et al., 2002). The attachment strategies of epiphytes vary according to the microstructure of the substrate. On smooth, flat host surfaces, root hairs flatten to increase contact area and secrete mucilaginous substances to enhance adhesion. On rough host surfaces, root hairs adopt tubular forms that conform to microcracks in the substrate, achieving attachment through mechanical interlocking between the root hairs and the substrate (Tay et al., 2022). Climbing plants generally prefer supports with rough surfaces, exhibiting higher attachment success on trees with coarse bark (Wu et al., 2016; Wang Z. W. et al., 2022; Yuan et al., 2010). However, excessively rough substrates may reduce effective contact, thereby decreasing attachment strength (Tay et al., 2022). For instance, in *Syngonium podophyllum*, the number and morphology of its aerial roots are controlled by environmental factors. Although adventitious roots can grow normally on a variety of organic and inorganic host surfaces, aerial roots exhibit significantly greater elongation when climbing on wooden surfaces. In contrast, plants growing on brick walls show significantly higher stem and petiole biomass (Wang et al., 2024). This indicates that different host surface materials have a significant impact on the growth of shade-tolerant root-attaching aroid vines.

Beyond surface roughness, host characteristics such as stability, bark shedding, water retention, and branch turnover rate significantly influence the long-term growth and development of climbing plants (Tay et al., 2023; Duarte and Gandolfi, 2017). Additionally, some studies have suggested that the growth of climbing stems in vines responds to the color and proximity of the support material (Price and Wilcut, 2007; Guerra et al., 2019), and leaf damage can accelerate the vine’s ability to twist around the support (Atala and Gianoli, 2008). However, research on the response of aroid vines, especially on their reaction to the characteristics of host surfaces, remains relatively underdeveloped.

### 5.3 Mechanical contact and growth direction

The growth strategies of climbing plants vary depending on the support conditions.Plants that secure vertical support exhibit higher light interception, biomass accumulation, photosynthetic productivity, and reproductive capacity (Mantuano et al., 2021). Vertical support entails maintaining mechanical contact with the substrate and adopting an upward growth orientation. Such mechanical contact triggers a suite of anatomical, physiological, biochemical, biophysical, and molecular responses collectively termed “thigmomorphogenesis” (Chehab et al., 2009; Li and Gong, 2011). Many aroid vines are highly sensitive to mechanical contact, such as *Monstera obliqua* and *Philodendron hederaceum* (Steinitz and Hagiladi et al., 1987). Under mechanical contact, aroid vines show increased biomass in the aerial parts, produce longer aerial roots, and their main stems elongate (Wang et al., 2024). For instance, in potted *Epipremnum aureum*, vertical support significantly increases leaf area compared to hanging plants (Di Benedetto et al., 2010). When climbing plants fail to attach and instead grow horizontally, they stimulate the growth of lateral branches and creeping stems. The creeping stems will crawl along the ground and develop searching shoots to locate supports. This pattern of growth allows the plant to continue growing and increases its chances of finding support (Den Dubbelden, 1994). In both creeping and hanging growth, the plant reduces its investment in the aerial parts (such as stems, leaves, and aerial roots), slowing down growth, while increasing investment in the underground roots to enhance absorption and anchorage (Wyka et al., 2019).

The direction of growth significantly influences the morphology and physiological characteristics of aroid vines. This type of response is generally referred to as “gravitropic morphogenesis,” which is associated with the polarity and distribution of auxin. The polar transport of auxins from the shoot tip to the root tip is often inhibited when the stem is hanging or growing horizontally. In hanging stems, auxins accumulate near the tip, which could ultimately lead to reduced growth of the whole plant (Lovisolo et al., 2002; Keller, 2015). Studies show that the net assimilation rate and photosynthetic rate of climbing *Epipremnum aureum* are higher than in creeping or hanging plants, with relative growth rates declining as the plant deviates from the vertical orientation. Exogenous auxins can promote an increase in leaf area and total leaf area in creeping and hanging plants, but they may reduce the relative extension rate, net assimilation rate, and relative growth rate in climbing plants. This indicates that non-vertical growing aroid vines suffer from insufficient endogenous hormones due to the suppression of auxin transport in their growth direction, while exogenously applying hormones to climbing plants may lead to an overdose of auxins, thereby inhibiting growth (Di Benedetto et al., 2018). Recent research on identifying the components responsible for mechanical and gravity responses, such as genes, proteins, hormones, and inorganic signaling molecules, has made progress (Coutand and Mitchell, 2016). Thigmomorphogenesis is regulated by hormone homeostasis, particularly the distribution of auxins.

Both gravitropic morphogenesis and thigmomorphogenesis likely influence the growth of the plant simultaneously. Steinitz (Steinitz et al., 1992) reported the interaction between the direction of *Epipremnum aureum*’s vines and their response to contact stimuli, showing that only upward-growing vines are sensitive to touch. Brito (Brito et al., 2022) tested the simultaneous interaction of light intensity, growth direction, and contact effects on Epipremnum aureum, demonstrating that the large increase in leaf area to form heteromorphic leaves was a synergistic effect of the application of light, growth direction, and mechanical contact.

## 6 Research prospects

### 6.1 Environmental adaptation and morphological plasticity strategies of aroid vines

Aroid vines are predominantly distributed in tropical rainforests and exhibit negative phototropism, with a strong dependency on high humidity and stable temperatures. Current research needs to further quantify the physiological thresholds of these plants, analyze their molecular response strategies, and clarify the hormonal regulatory networks in different light, temperature, and humidity environments, as well as their effects on stomatal opening and transpiration efficiency. Exploring the synergistic strategies between aroid vines and their microenvironments will help understand how environmental gradient changes influence their epiphytic traits and attachment efficiency, thus identifying optimal conditions for their support structures in terms of light, temperature, and humidity.

Aroid vines exhibit various life forms, including isomorphic, allomorphic, and heteromorphic types, with the characteristic of heterophylly. Their leaf morphology can adjust with climbing height, and they also demonstrate differences between climbing and creeping stems, as well as underground roots and aerial roots. These plants show varying resource allocation strategies at different growth stages. Future research should delve deeper into the environmental signaling pathways, exploring how light, humidity, contact, and CO_2_ concentration affect morphological development and the relationship between climbing height and plant longevity. Models of growth and hormone regulation networks should be established to explore how different hormones influence these morphological changes, facilitating the rapid formation of mature forms for landscaping applications.

### 6.2 Response of aroid vines to host surface characteristics and landscape applications

The physical properties and spatial structure of the support material significantly affect the climbing efficiency and morphological plasticity of vines. However, there is limited research on how aroid vines respond to different support characteristics. Future studies can focus on factors such as the roughness, color, water retention, and proximity of the support to quantify their influence on the climbing behavior of aroid vines. Understanding how aroid vines utilize resources in different states of growth and developing biomimetic climbing materials will help create low-cost, durable structures for urban vertical greening, improving the attachment efficiency of aroid vines in city applications.

Aroid vines hold significant potential for vertical greening applications. In landscape practice, they should be provided with rough, stable, and water-retentive supports to ensure effective contact and an upward growth orientation. During the later stages of climbing, adequate light and sustained connections of adventitious roots to the soil should be ensured to support continued growth. To promote further application, careful species selection and planting configuration are necessary. Different greening objectives should be matched with appropriate coverage and aesthetic effects. For example, at Singapore’s Changi Airport, a water-recirculation system was integrated with multiple shade-tolerant aroid lianas combined with diverse woody plants, creating a visual focal point in the Rain Vortex waterfall while enhancing the local microclimate. Based on this, exploring the relationship between pruning and coverage rate will improve the branching problem of aroid vines. Developing plant growth models, using 3D modeling techniques to predict plant coverage dynamics, and employing micro-environmental regulation technologies such as humidity control and shading systems will help simulate their natural habitat conditions. Evaluating the ecological services, such as cooling, humidifying, air purification, and supporting biodiversity, will be crucial for advancing their use in urban landscapes.

It is worth noting that a small number of aroid vines are recognized as potentially invasive species, such as *Epipremnum pinnatum* (Global Invasive Species Database, 2025). In tropical forests lacking strong competitors, these species can reproduce vegetatively to form dense populations, displacing native vegetation. Therefore, in horticultural and landscape applications, cultivars with invasive potential should be avoided in native conservation forests or ecologically fragile areas. In addition, some aroid vines exhibit a degree of toxicity. While casual contact poses no significant risk to humans, exposure to their sap may cause skin irritation, and ingestion could lead to poisoning. In landscape practices, clear labeling should be provided to prevent accidental ingestion or skin contact.

## 7 Conclusion

In conclusion, aroid vines are widely distributed and exhibit distinct tropical distribution patterns, with unique climbing strategies and growth traits characteristic of passive climbers. Based on life form, they can be classified as terrestrial vines, epiphytic vines, and semi-epiphytic vines; based on the degree of leaf heteromorphy, they can be classified as isomorphic vines, allomorphic vines, and heteromorphic vines. Their core climbing strategy relies on root attachment, and their aerial roots exhibit dimorphism. These plants utilize creeping stems to search for supports and employ clasping roots for attachment. During the climbing process, to cope with environmental changes from ground-level creeping to canopy ascent, aroid vines exhibit strong morphological plasticity. Resource allocation is biased toward the aboveground parts, ensuring hydraulic supply through increased feeder roots production, stem thickening, and hydraulic optimization. Concurrently, the development of larger and thicker heterophyllous leaves enhances photosynthetic capacity and drought tolerance, adapting to the high-light conditions of the canopy. Multiple factors influence the climbing growth of aroid vines: negative phototropism drives the search for supports, light intensity directly regulates leaf morphological development, and host surface traits, such as surface roughness and water retention significantly affect attachment efficiency and biomass allocation. Mechanical contact and gravity direction influence growth through hormone homeostasis, coordinating resource distribution. However, current research still faces gaps, such as a lack of field-based life form classification and unquantified support response strategies. The molecular networks of environmental adaptation and hormone regulation pathways remain to be elucidated. Future research should focus on the environmental adaptability and morphological plasticity strategies of aroid vines, their response to host surface characteristics, and their landscape applications.

## References

[B1] Ardiningtyas S. A. Matra D. D. Poerwanto R. Krisantini Asih N. P. S. (2023). Morphology study, leaf anatomy and growth of the Indonesian *Scindapsus*. J. Appl. Horticult. 25, 281–285. doi: 10.37855/jah.2023.v25i03.50

[B2] Atala C. Gianoli E. (2008). Induced twining in Convolvulaceae climbing plants in response to leaf damage. Botany 86, 595–602. doi: 10.1139/b08-037

[B3] Boyce P. C. Croats T. B . (2020). Data from: The Uberlist of Araceae Totals for Published and Estimated Number of Species in Aroid Genera. ResearchGate. doi: 10.13140/RG.2.2.24820.09605

[B4] Boyce P. C. Wong S. Y. (2009). The Aroids of the West Sarawak Limestone. IAS Newslett. 31, 1–8. Available online at: https://ir.unimas.my/id/eprint/7265 (Accessed October 14, 2025).

[B5] Boyce P. C. Wong S. Y. (2019). Borneo and its disproportionately large rheophytic aroid flora. Gardens’ Bull. Singapore 71, 497–524. doi: 10.26492/gbs71(suppl.2).2019-24

[B6] Brito C. Mantuano D. De Toni K. L. G. Mantovani A. (2022). Untangling leaf expansion triggers: A new experimental study with *Epipremnum aureum* (Araceae). Flora. 295, 152139. doi: 10.1016/j.flora.2022.152139

[B7] Cai Y. L. Song Y. C. (2001). Adaptive characteristics and behavior of Dalbergia millettii liana in subtropical evergreen broad-leaved forest of eastern China. Acta Ecol. Sinica 21, 216–224. doi: 10.3321/j.issn:1000-0933.2001.02.007

[B8] Callaway R. M. Reinhart K. O. Moore G. W. Moore D. J. Pennings S. C. (2002). Epiphyte host preferences and host traits: Mechanisms for species-specific interactions. Oecologia 132, 221–230. doi: 10.1007/s00442-002-0968-2, PMID: 28547355

[B9] Chatakul P. Janpathompong S. (2022). Interior plants: Trends, species, and their benefits. Building Environment 222, 109325. doi: 10.1016/j.buildenv.2022.109325

[B10] Chehab E. W. Eich E. Braam J. (2009). Thigmomorphogenesis: a complex plant response to mechano-stimulation. J. Exp. Botany 60, 43–56. doi: 10.1093/jxb/ern289, PMID: 19088336

[B11] Collantes J. P. Chua-Mangussad V. Hein K. Z. Bustamante R. A. A. (2024). *Homalomena brachygyna* (Araceae), a new species from Palawan Island, Philippines. Phytotaxa. 664, 59–67. doi: 10.11646/phytotaxa.664.1.5

[B12] Coutand C. Mitchell S. J. (2016). Mechanical signalling in plants: from perception to consequences for growth and morphogenesis (thigmomorphogenesis) and ecological significance. Front. Plant Sci. 7. doi: 10.3389/fpls.2016.01441, PMID: 27766100 PMC5052320

[B13] Croat T. B. (1979). The distribution of the Araceae (London: Academic Press).

[B14] Croat T. B. (1988). The ecology and life forms of Araceae. Aroideana – J. Int. Aroid Soc. 11, 4–56.

[B15] Croat T. B. (1992). Species diversity of Araceae in Colombia: A preliminary survey. Ann. Missouri Botanical Garden 79, 17–28. doi: 10.2307/2399806

[B16] Croat T. B. (1995). “ Floristic comparisons of Araceae in six Ecuadorian florulas,” in Biodiversity and Conservation of Neotropical Montane Forests. Eds. Churchill S. P. Balslev H. Forero E. Luteyn J. L. ( he New York Botanical Garden, New York, NY), 489–499.

[B17] Croat T. B. Ortiz O. O. (2020). Distribution of Araceae and the Diversity of Life Forms. Acta Societatis Botanicorum Poloniae 89, 8939. doi: 10.5586/asbp.8939

[B18] Cusimano N. Bogner J. Mayo S. J. Boyce P. C. Wong S. Y. Hesse M. . (2011). Relationships within the Araceae: Comparison of morphological patterns with molecular phylogenies. Am. J. Botany 98, 654–668. doi: 10.3732/ajb.1000158, PMID: 21613165

[B19] Darwin C. (1865). On the Movements and Habits of Climbing Plants. Linn. Soc. Trans. 9, 111–118. doi: 10.1111/j.1095-8339.1865.tb00011.x

[B20] Den Dubbelden K. C. (1994). Growth and allocation patterns in herbaceous climbing plants (Utrecht: Utrecht University Press).

[B21] Díaz-Valverde V. Avalos G. Quesada-Fonseca J. (2025). Phenotypic differences in sun and shade leaves of *Monstera deliciosa* (Araceae). Rev. Biología Trop. 73, 56794. doi: 10.15517/rev.biol.trop.v73i1.56794

[B22] Di Benedetto A. Galmarini C. Tognetti J. (2018). New insight into how thigmomorphogenesis affects *Epipremnum aureum* plant development. Horticult. Brasileira 36, 330–340. doi: 10.1590/s0102-053620180307

[B23] Di Benedetto A. Tognetti J. Galmarini C. (2010). Biomass production in ornamental foliage plants: Crop productivity and mechanisms associated to exogenous cytokinin supply. Am. J. Plant Sci. Biotechnol. 4, 1–22.

[B24] Duarte M. M. Gandolfi S. (2017). Diversifying growth forms in tropical forest restoration: Enrichment with vascular epiphytes. For. Ecol. Manage. 401, 89–98. doi: 10.1016/j.foreco.2017.06.063

[B25] Durigon J. Durán S. M. Gianoli E. (2013). Global distribution of root climbers is positively associated with precipitation and negatively associated with seasonality. J. Trop. Ecol. 29, 357–360. doi: 10.1017/S0266467413000308

[B26] Einzmann H. J. R. Weichgrebe L. Kohlstruck J. Zotz G. (2024). Climbing aroids in a Panamanian lowland forest: We should reconsider our categories. J. Vegetation Sci. 35, e13246. doi: 10.1111/jvs.13246

[B27] Filartiga A. L. Mantuano D. Vieira R. C. De Toni K. L. G. Vasques G. M. Mantovani A. (2021). Root morphophysiology changes during the habitat transition from soil to canopy of the aroid vine *Rhodospatha oblongata*. Ann. Botany 127, 347–360. doi: 10.1093/aob/mcaa182, PMID: 33038225 PMC7872123

[B28] Filartiga A. L. Vieira R. C. Mantovani A. (2014). Size−correlated morphophysiology of the aroid vine *Rhodospatha oblongata* along a vertical gradient in a Brazilian rain forest. Plant Biol. 16, 155–165. doi: 10.1111/plb.12023, PMID: 23614870

[B29] Filartiga A. L. Vieira R. C. Mantovani A. (2018). Aerial root hydraulic conductivity increases with plant size for the aroid vine *Rhodospatha oblongata* (Araceae). J. Plant Hydraulics 4, e006. doi: 10.20870/jph.2017.e006

[B30] French J. C. (1987). Systematic occurrence of a sclerotic hypodermis in roots of Araceae. Am. J. Botany 74, 891–903. doi: 10.1002/j.1537-2197.1987.tb08693.x

[B31] Fritz M. A. Rosa S. Sicard A. (2018). Mechanisms underlying the environmentally induced plasticity of leaf morphology. Front. Genet. 9. doi: 10.3389/fgene.2018.00025, PMID: 30405690 PMC6207588

[B32] Global Invasive Species Database (2025). Species profile: *Epipremnum pinnatum*. Available online at: https://www.iucngisd.org/gisd/speciesname/Syngonium+podophyllum (Accessed October 14, 2025).

[B33] Guerra S. Peressotti A. Peressotti F. Bulgheroni M. Baccinelli W. D’Amico E. (2019). Flexible control of movement in plants. Sci. Rep. 9, 16570. doi: 10.1038/s41598-019-53118-0, PMID: 31719580 PMC6851115

[B34] Henny R. J. Norman D. J. Chen J. (2004). Progress in ornamental aroid breeding research. Ann. Missouri Botanical Garden 91, 464–472. doi: 10.2307/3298535

[B35] Henriquez C. L. Arias T. Pires J. C. Croat T. B. Schaal B. A. (2014). Phylogenomics of the plant family Araceae. Mol. Phylogenet. Evol. 75, 91–102. doi: 10.1016/j.ympev.2014.02.017, PMID: 24594061

[B36] Herrera F. A. Jaramillo C. A. Dilcher D. L. Wing S. L. Gómez-N C. (2008). Fossil Araceae from a Paleocene Neotropical Rainforest in Colombia. Am. J. Botany 95, 1569–1583. doi: 10.3732/ajb.0800172, PMID: 21628164

[B37] Holtum J. A. M. Winter K. Weeks M. A. Sexton T. R. (2007). Crassulacean acid metabolism in the ZZ plant, *Zamioculcas zamiifolia* (Araceae). Am. J. Bot. 94, 1670–1676. doi: 10.3732/ajb.94.10.1670, PMID: 21636363

[B38] International Aroid Society (2025). Genera. Available online at: https://www.aroid.org/genera (Accessed October 14, 2025).

[B39] Isnard S. Silk W. K. (2009). Moving with climbing plants from Charles Darwin’s time into the 21st century. Am. J. Botany 96, 1205–1221. doi: 10.3732/ajb.0900045, PMID: 21628270

[B40] Jia X. B. Wei J. Q. Chen Y. W. Zeng C. H. Deng C. Zeng P. C. . (2025). Codon usage patterns and genomic variation analysis of chloroplast genomes provides new insights into the evolution of Aroideae. Sci. Rep. 15, 1433. doi: 10.1038/s41598-025-88244-5, PMID: 39910236 PMC11799533

[B41] Kato S. (2019). “ Assessing negative and positive phototropism in lianas,” in Phototropism: Methods and Protocols. Ed. Yamamoto K. ( Humana Press, New York), 19–26., PMID: 10.1007/978-1-4939-9015-3_2 30694463

[B42] Kato S. Kanematsu T. Kawakubo N. Komiyama A. (2012a). Positive and negative phototropism in *Schizophragma hydrangeoides* and *Parthenocissus tricuspidata*. Japanese J. For. Environment 54, 1–5. doi: 10.18922/jjfe.54.1_1

[B43] Kato S. Yamamoto T. Kawakubo N. Komiyama A. (2012b). Responses of *Trachelospermum asiaticum* (Apocynaceae) seedlings to growth in a light intensity gradient. Ecol. Res. 27, 229–231. doi: 10.1007/s11284-011-0871-y

[B44] Keller M. (2015). *The science of grapevines: Anatomy and physiology* (2nd ed.) (London: Academic Press).

[B45] Kress W. J. (1986). The systematic distribution of vascular epiphytes: an update. Selbyana 9, 2–22. doi: 10.1111/boj.12010

[B46] Li Z. G. Gong M. (2011). Mechanical stimulation-induced cross-adaptation in plants: An overview. J. Plant Biol. 54, 358–364. doi: 10.1007/s12374-011-9101-6

[B47] Liang A. Sima Y. K. Zhang X. Xu T. (2019). The Geographical Distribution of the Endemic Species of Araceae in China. Chin. Wild Plant Resources 38, 77–85. doi: 10.3969/j.issn.1006-9690.2019.06.015

[B48] Liu F. Liu J. Dong M. (2016). Ecological consequences of clonal integration in plants. Front. Plant Sci. 7. doi: 10.3389/fpls.2016.00770, PMID: 27446093 PMC4927562

[B49] López−Portillo J. Ewers F. W. Angeles G. Fisher J. B. (2000). Hydraulic architecture of *Monstera acuminata*: evolutionary consequences of the hemiepiphytic growth form. New Phytol. 145, 289–299. doi: 10.1046/j.1469-8137.2000.00578.x

[B50] Lorenzo L. Mantuano D. Mantovani A. (2009). Comparative leaf ecophysiology and anatomy of seedlings, young and adult individuals of the epiphytic aroid *Anthurium scandens* (Aubl.) Engl. Environ. Exp. Bot. 68, 314–322. doi: 10.1016/j.envexpbot.2009.11.011

[B51] Loss-Oliveira L. Sakuragui C. Soares M. D. L. Schrago C. G. (2016). Evolution of *Philodendron* (Araceae) species in Neotropical biomes. PeerJ. 4, e1744. doi: 10.7717/peerj.174410.7717/peerj.1744, PMID: 27042390 PMC4811177

[B52] Lovisolo C. Schubert A. Sorce C. (2002). Are xylem radial development and hydraulic conductivity in downwardly-growing grapevine shoots influenced by perturbed auxin metabolism? New Phytol. 156, 65–74. doi: 10.1046/j.1469-8137.2002.00405.x, PMID: 12817565

[B53] Mantovani A. Brito C. Mantuano D. (2018). Does the same morphology mean the same physiology? Morphophysiological adjustments of *Philodendron hederaceum* (Jacq.) Schott, an isomorphic aroid, to ground–canopy transition. Theor. Exp. Plant Physiol. 30, 89–101. doi: 10.1007/s4062601801056

[B54] Mantovani A. Mantuano D. de Mattos E. A. (2017). Relationship between nitrogen resorption and leaf size in the aroid vine *Rhodospatha oblongata* (Araceae). Aust. J. Botany 65, 431–437. doi: 10.1071/BT16231

[B55] Mantuano D. Ornellas T. Aidar M. P. M. (2021). Photosynthetic activity increases with leaf size and intercellular spaces in an allomorphic lianescent aroid *Rhodospatha oblongata* (Araceae). Funct. Plant Biol. 48, 557–566. doi: 10.1071/FP20215, PMID: 33556303

[B56] Meng X. Yan L. Y. Liu F. D. (2022). A new method to improve indoor environment: Combining the living wall with air-conditioning. Building Environ. nt. 26, 108981. doi: 10.1016/j.buildenv.2022.108981

[B57] Metcalfe D. J. (2005). Biological flora of the British Isles: *Hedera helix* L. J. Ecol. 93, 632–648. doi: 10.1111/j.1365-2745.2005.01021.x

[B58] Moffett M. W. (2000). What’s “Up”? A critical look at the basic terms of canopy biology. Biotropica 32, 569–596. doi: 10.1646/0006-3606(2000)032[0569:WSUACL]2.0.CO;2

[B59] Moodley D. Procheş Ş. Wilson J. R. U. (2017). Assessing and managing the threat posed by Epipremnum aureum in South Africa. South Afr. J. Botany 109, 178–188. doi: 10.1016/j.sajb.2016.12.005

[B60] Mou F. J. Li Y. G. Wang C. M . (2019). Liana Plant Resources (Volume 1) (Beijing: Science Press).

[B61] Nakayama H. (2024). Leaf form diversity and evolution: a never-ending story in plant biology. J. Plant Res. 137, 547–560. doi: 10.1007/s10265024015414, PMID: 38592658 PMC11230983

[B62] Othman A. Boyce P. C. Keng C. L. (2010). Studies on Monstereae (Araceae) of Peninsular Malaysia III: *Scindapsus lucens*, a new record for Malaysia. Garden’s Bull. Singapore 21, 1–6. PMC3819072 24575195

[B63] Paul G. S. Yavitt J. B. (2011). Tropical vine growth and the effects on forest succession: A review of the ecology and management of tropical climbing plants. Botanical Rev. 77, 11–30. doi: 10.1007/s12229-010-9059-3

[B64] Poorter L. Rozendaal D. M. A. (2008). Leaf size and leaf display of thirty-eight tropical tree species. Oecologia. 158, 35–46. doi: 10.1007/s00442-008-1115-1, PMID: 18719946

[B65] Price A. J. Wilcut J. W. (2007). Response of ivyleaf morningglory (*Ipomoea hederacea*) to neighboring plants and objects. Weed Technol. 21, 922–927. doi: 10.1614/WT-06-146.1

[B66] Putz F. E. (1984). The natural history of lianas on Barro Colorado Island, Panama. Ecology. 65, 1713–1724. doi: 10.2307/1937767

[B67] Putz F. E. Holbrook N. M. (1986). Notes on the natural history of hemiepiphytes. Selbyana 9, 61–69.

[B68] Putz F. E. Mooney H. A. (2011). The Biology of Vines (Cambridge: Cambridge University Press).

[B69] Ray T. S. (1981). Growth and heterophylly in an herbaceous vine, *Syngonium* (Araceae) (Cambridge (MA: Harvard University).

[B70] Ray T. S. (1986). Growth correlations within the segment in the Araceae. Am. J. Botany 73, 993–1001. doi: 10.1002/j.1537-2197.1986.tb08543.x

[B71] Ray T. S. (1987). Cyclic heterophylly in *Syngonium* (Araceae). Am. J. Botany 74, 16–26. doi: 10.1002/j.1537-2197.1987.tb08575.x

[B72] Ray T. S. (1989). Diversification of growth habits in the Araceae. Am. J. Botany 76, 267. doi: 10.2307/2444571

[B73] Ray T. S. (1990). Metamorphosis in the Araceae. Am. J. Botany 77, 1599–1609. doi: 10.1002/j.1537-2197.1990.tb11400.x

[B74] Rodriguez–Quintero W. D. Moreno−Chacón M. Saldaña A. Knapik H. Gianoli E. (2022). From dark to darkness, negative phototropism influences the support−tree location of the massive woody climber *Hydrangea serratifolia* (Hydrangeaceae) in a Chilean temperate rainforest. Plant Signaling Behavior. 17, 2122244. doi: 10.1080/15592324.2022.2122244, PMID: 36476262 PMC9733698

[B75] Saibeh K. (2010). A numerical analysis of morphological and anatomical characteristics in Malaysian species of the aroid genus *Scindapsus Schott*. Borneo Sci. J. Sci. Technol. 26, 1–10.

[B76] Schnitzer S. A. Bongers F. (2002). The ecology of lianas and their role in forests. Trends Ecol. Evol. 17, 223–230. doi: 10.1016/S0169-5347(02)02491-6

[B77] Schnitzer S. A. Michel N. L. Powers J. S. Robinson W. D. (2020). Lianas maintain insectivorous bird abundance and diversity in a neotropical forest. Ecology 101, e03176. doi: 10.1002/ecy.3176, PMID: 32870500

[B78] Shiodera S. Rahajoe J. S. Kohyama T. (2008). Variation in longevity and traits of leaves among co-occurring understorey plants in a tropical montane forest. J. Trop. Ecol. 24, 121–133. doi: 10.1017/S0266467407004725

[B79] Smithsonian National Museum of Natural History . (2025). Lianas and Climbing Plants of the Neotropics: ARACEAE. Available online at: https://naturalhistory.si.edu/sites/default/files/media/file/araceae_0.pdf (Accessed October 16, 2025).

[B80] Sperotto P. Acevedo-Rodríguez P. Vasconcelos T. N. C. Roque N. (2020). Towards a standardization of terminology of the climbing habit in plants. Botanical Rev. 86, 180–210. doi: 10.1007/s12229-020-09218-y

[B81] Steinitz B. Hagiladi A. (1987). Thigmomorphogenesis in climbing *Epipremnum aureum*, *Monstera obliqua* ‘expilata’, and *Philodendron scandens* (Araceae). J. Plant Physiol. 128, 461–466. doi: 10.1016/S0176-1617(87)80114-6

[B82] Steinitz B. Hagiladi A. Anav D. (1992). Thigmomorphogenesis and its interaction with gravity in climbing plants of *Epipremnum aureum*. J. Plant Physiol. 140, 571–574. doi: 10.1016/S0176-1617(11)81743-7

[B83] Strong D. R. Ray T. S. (1975). Host tree location behavior of a tropical vine (*Monstera gigantea*) by skototropism. Science. 190, 804–806. doi: 10.1126/science.190.4216.804

[B84] Tay J. Y. L. Kovalev A. Zotz G. Einzmann H. J. R. Gorb S. N. (2022). Holding on or falling off: The attachment mechanism of epiphytic *Anthurium obtusum* changes with substrate roughness. Am. J. Bot. 109, 874–886. doi: 10.1002/ajb2.16000, PMID: 35608083

[B85] Tay J. Y. L. Zotz G. Einzmann H. J. R. (2023). Smoothing out the misconceptions of the role of bark roughness in vascular epiphyte attachment. New Phytol. 238, 983–994. doi: 10.1111/nph.18811, PMID: 36775857

[B86] Valadares R. T. Couto D. R. Manhaes V. D. Silva L. D. Dutra V. F. (2024). *Anthurium capixaba* (Araceae): a new species with cordate leaves from Brazil. Phytotaxa. 664, 75–82. doi: 10.11646/phytotaxa.664.1.7

[B87] Vasconcelos S. Soares M. L. Sakuragui C. M. Croat T. B. Oliveira G. Benko-Iseppon A. M. (2018). New insights on the phylogenetic relationships among the traditional *Philodendron* subgenera and the other groups of the *Homalomena* clade (Araceae). Mol. Phylogenet. Evol. 127, 168–178. doi: 10.1016/j.ympev.2018.05.017, PMID: 29787799

[B88] Verbeeck H. De Deurwaerder H. P. T. Kearsley E. Parvathi S. K. M. Mundondo F. M. Coppieters K. . (2024). Towards a liana plant functional type for vegetation models. Ecol. Model. 498, 110901. doi: 10.1016/j.ecolmodel.2024.110901, PMID: 39619676 PMC11568259

[B89] Wang P. Abid M. A. Qanmber G. Hussain M. Ahmad N. Ali S. . (2022). Photomorphogenesis in plants: the central role of phytochrome interacting factors (PIFs). Environ. Exp. Botany 194, 104704. doi: 10.1016/j.envexpbot.2021.104704

[B90] Wang Z. Z. Fan T. F. Weng S. F. (2024). Effects of Drought Stress and Climbing Surfaces on Growth of 2 Adhering Vines. J. Southwest Forestry University(Natural Sciences). 44, 9–17. doi: 10.11929/j.swfu.202310050

[B91] Wang J. Mo Q. F. Chu S. S. Lai C. Zeng S. C. (2018). Effects of sewage sludge compost on the growth and heavy metal accumulation in landscape plant *Syngonium podophyllum*. Chin. J. Ecol. 37, 1752–1758. doi: 10.13292/j.1000-4890.201806.001

[B92] Wang Z. W. Shang Q. Liu Y. C. (2022). Attachment Rules of Lianas on Trunks at Different Positions in Mixed Broadleaf-conifer Forest at Jigong Mountain. J. Trop. Subtropical Bot. 30, 492–499. doi: 10.11926/jtsb.4486

[B93] Ward E. B. Pregitzer C. C. Kuebbing S. E. Bradford M. A. (2020). Invasive lianas are drivers of and passengers to altered soil nutrient availability in urban forests. Biol. Invasions 22, 935–955. doi: 10.1007/s10530-019-02134-2

[B94] West G. B. Brown J. H. Enquist B. J. (1999). A general model for the structure and allometry of plant vascular systems. Nature 400, 664–667. doi: 10.1038/23251

[B95] Wu Z. Y. (2010). Flora of China, Volume 23 (English Edition) (Beijing: Science Press).

[B96] Wu H. D. Liu Q. Tan Y. H. Zhang J. L. (2016). Liana Diversity and Its Relationship with Host Trees in the Yuanjiang Dry-Hot Valley,Yunnan,China. Plant Sci. J. 34, 547–554. doi: 10.11913/PSJ.2095-0837.2016.40547

[B97] Wyka T. P. (2023). Negative phototropism of the shoots helps temperate liana *Hedera helix* L. @ to locate host trees under habitat conditions. Tree Physiol. 43, 1874–1885. doi: 10.1093/treephys/tpad077, PMID: 37334935

[B98] Wyka T. P. Zadworny M. Mucha J. Holtta T. Brezdeva M. Mencuccini M. . (2019). Species-specific responses of growth and biomass distribution to trellis availability in three temperate lianas. Trees. 33, 921–932. doi: 10.1007/s00468-018-1762-6

[B99] Xu Y. K. Kou P. W. Liu C. L. Li S. Sun X. C. Huang W. J. (2024). Structural Characteristics and Phylogenetic Analysis of Chloroplast Genomes in Araceae Plants: Take 20 Species such as Arisaema heterophyllum for Example. Chin. Wild Plant Resources 43, 42–50. doi: 10.3969/j.issn.1006-9690.2024.01.005

[B100] Yang X. Deng W. (2017). Morphological and structural characterization of the attachment system in aerial roots of *Syngonium podophyllum*. Planta. 245, 507–521. doi: 10.1007/s00425-016-2621-4, PMID: 27888361

[B101] Yang S. N. Shen W. J. Zhang L. (2018). Effects of Different Light Quality Ratios on the Morphogenesis of *Philodendron* ‘con-go’ Plantlets. Mol. Plant Breed. 16, 7171–7178. doi: 10.13271/j.mpb.016.007171

[B102] Yuan C. M. Liu W. Y. Yang G. P. Li X. S. (2010). Liana Species Diversity and Relationships with Its Host Trees in the Moist Evergreen Broad-Leaved Forest in the Ailao Mountains,Southwest China. Sci. Silvae Sinicae 46, 15–22. doi: 10.11707/j.1001-7488.20100103

[B103] Zhang G. Li H. Meng Y. J. Ge S. J. (2017). Research Progress on Germplasm Resources of *Arisaema erubescens*. J. Chin. Med. Mater. 40, 237–241. doi: 10.13863/j.issn1001-4454.2017.01.054

[B104] Zhang L. Yue J. J. Zhang J. (2018). Research Advance of Phototropism in Plants. Mol. Plant Breed. 16, 2015–2027. doi: 10.13271/j.mpb.016.002015

[B105] Zhao L. Yang Y. Y. Qu X. J. Ma H. Hu Y. Li H. T. . (2023). Phylotranscriptomic analyses reveal multiple whole-genome duplication events, the history of diversification and adaptations in the Araceae. Ann. Botany 131, 199–214. doi: 10.1093/aob/mcac062, PMID: 35671385 PMC9904356

[B106] Zimmermann M. H. (1983). Xylem structure and the ascent of sap (Berlin: Springer). 10.1126/science.222.4623.500-a 17746198

[B107] Zotz G. (2013). ‘Hemiepiphyte’: a confusing term and its history. Ann. Botany 111, 1015–1020. doi: 10.1093/aob/mct085, PMID: 23589630 PMC3662525

[B108] Zuluaga A. Llano M. Cameron K. (2019). Systematics, biogeography and morphological character evolution of the hemiepiphytic subfamily Monsteroideae (Araceae). Ann. Missouri Botanical Garden 104, 33–48. doi: 10.3417/2018269

